# Liver Cancer Neuroscience: Regulating Liver Tumors via Selective Hepatic Vagotomy

**DOI:** 10.3390/mps7060099

**Published:** 2024-12-11

**Authors:** Kylynda C. Bauer, Shadin Ghabra, Chi Ma, Lee Chedester, Tim F. Greten

**Affiliations:** 1 Thoracic and Gastrointestinal Malignancies Branch, Center for Cancer Research (CCR), National Cancer Institute (NCI), National Institutes of Health (NIH), Bethesda, MD 20892, USA; kylynda.bauer@nih.gov (K.C.B.); shadin.ghabra@medstar.net (S.G.); chi.ma2@nih.gov (C.M.); 2 Surgical Oncology Program, Center for Cancer Research (CCR), National Cancer Institute (NCI), National Institutes of Health (NIH), Bethesda, MD 20892, USA; 3 Division of Intramural Clinical and Biological Research, National Institute on Alcohol Abuse and Alcoholism, National Institutes of Health (NIH), Rockville, MD 20852, USA; leec@dicbr.niaaa.nih.gov; 4 Liver Cancer Program, Center for Cancer Research (CCR), National Cancer Institute (NCI), National Institutes of Health (NIH), Bethesda, MD 20814, USA

**Keywords:** A20, hepatic vagotomy, liver cancer, metastases, parasympathetic nervous system, vagus

## Abstract

Both the prevalence and mortality of liver cancers continue to rise. Early surgical interventions, including liver transplantation or resection, remain the only curative treatment. Nerves in the periphery influence tumor growth within visceral organs. Emerging cancer neuroscience efforts linked parasympathetic vagus nerves with tumor pathology, underscoring the value of vagal nerve denervation methods within cancer mouse models. Here, we describe a selective hepatic vagotomy that largely maintains non-liver parasympathetic innervation in mice. To address vagal interactions in hepatic tumor pathology, we provide an adapted methodology utilizing an established liver metastatic model. We anticipate that this methodology will expand the burgeoning field of cancer neuroscience, enabling the study of the neuroimmune, neurometabolic, and/or nerve–microbiota interactions shaping liver cancer progression and treatment.

## 1. Introduction

The liver—the largest internal mammalian organ—maintains systemic homeostasis by regulating diverse metabolic, digestive, and immune processes [1]. The central vein and portal triad, comprising the bile duct, hepatic artery, hepatic portal vein, and lymphatic vessels, facilitate hepatic circulation. Densely innervated, the liver filters gastrointestinal blood from portal vessels, while the hepatic artery carries oxygenated blood [1,2]. Another innervation track, however, often remains overlooked—the hepatic nervous system [3].

Peripheral fibers of the autonomic nervous system (ANS) accompany the portal triad forming a dynamic sensory arc capturing ionic, metabolic, and inflammatory hepatic cues [3,4]. The ANS comprises the enteric nervous system (ENS), as well as the complementary sympathetic (SNS) and parasympathetic (PSNS) nervous systems [5], regulating “fight-or-flight” and “rest-and-digest” processes, respectively [6]. Hepatic SNS fibers originate from celiac/superior mesenteric ganglia. SNS preganglionic release acetylcholine, while postganglionic fibers release nor/adrenaline. In contrast, the hepatic PSNS derives from the cholinergic vagus nerve [2,3,4,5,7].

The vagus nerve, cranial nerve X, is the largest ANS nerve, innervating many thoracic and abdominal organs [8]. Vagal fibers enter the liver via the common hepatic branch. While vagal fibers terminate in the surrounding hilar region and interface with bile ducts, most descend via the gastroduodenal sub-branch terminating in the antral stomach, duodenum, and pancreas [9,10,11]. Gastric and celiac branches of the vagus nerve provide additional PSNS input to the stomach, intestinal tract, and pancreas [11,12]. The hepatic vagal branch contributes to numerous liver functions, including hepatocyte regeneration [13], glucose metabolism [14], and systemic inflammation [15]. Disruption of vagal integrity alters metabolic and immune processes, likely modulating diverse liver diseases, including cancer [16,17,18]. 

Liver cancer remains a principal cause of cancer-related death worldwide [19,20]. Liver cancer incidence increased by 75% from 1990–2015 [21], with >1 million global cases estimated by 2025 [22]. Moreover, the liver is a key site for metastases with far more cases of metastases to the liver compared to primary tumor formation [23,24,25]. Recent studies linked PSNS signaling with tumor pathology in abdominal solid tumors [26,27]. While vagal nerves shape liver metabolic and inflammatory responses [4,13,14,28], their role in hepatic tumors remains largely unstudied.

A key technique to disrupt the PSNS is vagotomy—snipping of the vagal nerve. Within clinical settings, vagotomy has been utilized to treat peptic ulcers, although non-surgical pharmacological approaches are the current standard of care [29,30]. A 2021 clinical study following ~50,000 patients that underwent peptic ulcer surgical procedures reported a significantly reduced risk of hepatobiliary cancers in patients that underwent surgeries including vagotomy [31], while ongoing work by our group showed that prior vagal disruption controls subsequent primary liver tumor growth [18].

Hepatic vagotomy was previously offered as a commercial procedure (Charles River Laboratories, discontinued summer 2022). Here, we provide a detailed protocol of hepatic branch vagotomy and appropriate sham surgical controls. In addition, we provide techniques to perform vagotomies in mice exhibiting liver tumors (A20 metastatic model). This methodology was designed to assess hepatic PSNS disruption during cancer treatment studies and may be performed in conjunction with immunotherapy strategies. Final readouts can be assessed within a relatively short timeframe (2–4 weeks) to examine the underexplored nerve–liver axis in cancer.

## 2. Experimental Design

This method examines the impact of hepatic branch vagotomy in murine models of liver tumors. Non-selective and selective vagotomy procedures have been performed in mice, including celiac, cervical, and subdiaphragmatic [32,33,34,35]. A detailed description of various vagotomy options is depicted in Mastitskaya et al., 2016 [35]. Truncal (subdiaphragmatic) vagotomy, shearing the left and right vagal branch alongside the esophagus as it descends from the diaphragm, has long been utilized for relative ease of nerve isolation in rodent models, with subdiaphragmatic vagotomy methodology previously reported in mouse models of gastric [17,36], small intestinal [37], colorectal [38], and pancreatic cancer [39]. Significant denervation via subdiaphragmatic or bilateral cervical vagotomy eradicates PSNS signaling throughout the viscera and should be performed in conjunction with pyloroplasty to counter delayed gastric emptying [32,40]. Hepatic vagotomy, in contrast, provides a more targeted approach that largely maintains abdominal innervation. 

Here, we provide a variation of the technique in mice exhibiting liver tumors. We use an A20 B cell lymphoma cell line derived from BALB/c mice [41] and purchased from ATCC (American Type Culture Collection). Tumor establishment utilized an established liver metastasis model [42,43,44]. To maintain syngeneic transplantation, our studies were performed in BALB/cAnNCrl mice (Bagg-albino stock #028) purchased from Charles River Laboratories. Following tail vein injection, A20 cells travel into the liver via the portal vein and lodge within the hepatic parenchyma, recapitulating clinical metastatic formation. Macroscopic metastatic tumors form within two weeks. Animals were housed at the NIH Clinical Research Center Animal Facility (Bethesda, MD, USA). All housing, procedures, and experimental endpoints were performed in accordance with the NCI Institutional Animal Care and Use Committee following the National Research Council’s *Guide for the Care and Use of Laboratory Animals*. Animal welfare was assessed daily.

### 2.1. Materials

- 0.9% sterile saline solution (Quality Biological, Gaithersburg, MD, USA, catalogue #:114-055-101);
- 10% povidone–iodine solution (Dynarex catalogue #: 1425);
- 29 G, 0.3 mL BD insulin syringes with needle (BD Biosciences catalogue #: 324702);
- 70% ethanol (Sigma-Aldrich, Darmstadt, Germany, Millipore Sigma catalogue #: EX0281-1);
- Coated VICRYL™ 5-0 sutures (Johnson & Johnson, Neuchâtel, Switzerland, Ethicon catalogue #: J391H);
- Cotton-tipped applicators (Medline catalogue #: MDS202000);
- Lubricant PM Ointment (AACE Pharmaceuticals Fairfield, NJ, USA, catalogue #: 71406-124-35);
- Peri-operative analgesia;
- Sterile alcohol prep pads (Dynarex catalogue #: 116).
- ***A20 Cell Culture***
- 2-Mercaptoethanol (Sigma-Aldrich, Darmstadt, Germany, Millipore Sigma catalogue #: M6250-10ML);
- 4-(2-hydroxyethyl)-1-piperazineethanesulfonic acid (HEPES; ThermoFisher Scientific, Grand Island, NY, USA, catalogue #: 15630080);
- A20 cells (ATCC, Manassas, VA, USA, catalogue #: TIB-208);
- Fetal bovine serum (FBS; GeminiBio, West Sacramento, CA, USA catalogue #: 100-106);
- Minimal essential media non-essential amino acid solution (MEM-NEAA;
- ThermoFisher Scientific, Grand Island, NY, USA, catalogue #: 11140050);
- Sodium pyruvate (ThermoFischer Scientific, Grand Island, NY, USA, catalogue #: 11360070);
- Penicillin/streptomycin antibiotics (ThermoFisher Scientific, Grand Island, NY, USA, catalogue #: 15140122);
- Phosphate-buffered saline (PBS; ThermoFisher Scientific, Grand Island, NY, USA, catalogue #: 14190144);
- RPMI 1640 Gibco cell culture medium (ThemoFischer Scientific, Grand Island, NY, USA, catalogue #: 11875093).

### 2.2. Equipment

- ***Cell culture and preparation***
- 37° sterile incubator for cell culture;
- 4° refrigerator, ice/ice box;
- Centrifuge;
- Light microscope;
- Sterile laminar flow hood for tissue culture;
- Serological pipettes, pipette tips, and tissue culture flasks (T75);
- Sterile Eppendorf tubes, 15/50 mL conical tubes.
- ***Surgical Suite***
- Bead sterilizer;
- Fiberoptic lighting for surgical arena;
- Heating pad and/or heat pump and heat lamp;
- Isoflurane gas anesthesia system (cage and nose cone outputs) and oxygen tank with scavenging system;
- Postoperative rodent cages;
- Surgical drapes and sterile gowning/gloves.
- ***Surgical Instruments***
- 4.0× Surgical Loupes, 340 mm working distance (Ted Pella, Redding, CA, USA, catalogue #: 75426);
- 9 mm Autoclip Kit (Staples, Autoclip applier/remover; Braintree Scientific, Braintree, MA, USA catalogue #: NC9946451);
- Dumont forceps super fine tip #5SF (Fine Science Tools catalogue #: 11252-00);
- Dumont forceps micro-blunted tips #5 (Fine Science Tools catalogue #: 11253-20);
- Needle driver;
- Razor (electrical clipper for shaving mouse abdomen) or hair removal cream;
** Retractor: Kratz-Barraquer eye speculum (Bausch + Lomb catalogue #: E4107 K) or 6.5 mm × 16 mm solid blade retractor (Cooper Surgical catalogue #: 3338-4G) and blunt hook retractor (Volkman Hook; GerMedUSA, Garden City Park, NY, USA, catalogue #: G24-166);
- Straight or curved surgical scissors and forceps;
- Paper/surgical tape.
- Methodology can be adapted to any micro-forceps. We list recommended forceps within ***Surgical Instruments***.
** This methodology utilizes a Kratz-Barraquer-styled speculum retractor system and hook retraction. Charles River Laboratories utilized solid blade retractor set-up. Follow institutional protocols on abdominal retraction.

### 2.3. Optional

- Autoclave within rodent facility;
- Tabletop dissecting microscope;
- Dental LED light (increased lighting for surgical arena);
- Rodent ear tags/tattoo ink (identification marker);
- VetOne Silver Nitrate Applicators (to cauterize minor bleeding; VetDepot, catalogue #: 1050871).

**Figure 1:**
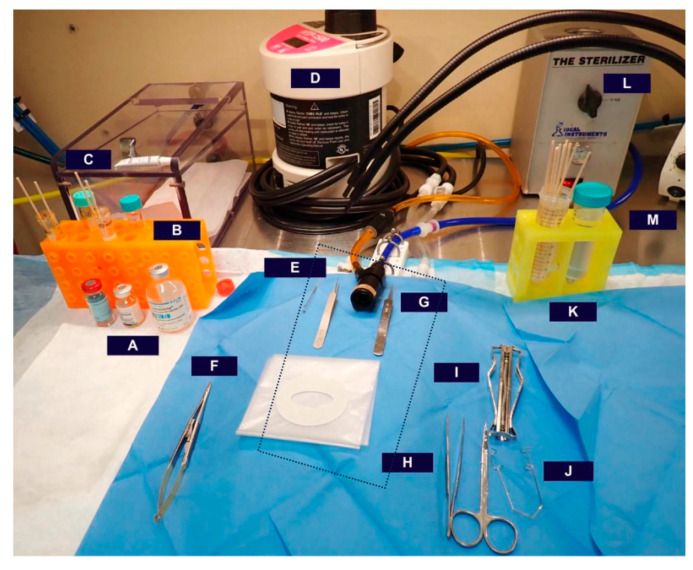
Example of surgical set-up in murine facility, with the dotted square indicating the heated surgical table under sterile draping, including a isoflurane nose cone: (**A**) perioperative surgical analgesic; (**B**) 15 mL test tubes containing either 10% povidone–iodine solution or 70% ethanol with cotton applicator tips; (**C**) lubricating gel to protect rodent eyes (pictured on top of the isoflurane induction chamber); (**D**) heating pump; (**E**) blunt hook retractor; (**F**) needle driver; (**G**) micro-forceps; (**H**) forceps/scissors; (**I**) staple applicator; (**J**) Kratz-Barraquer retractor; (**K**) 50 mL tubes of sterile saline, one containing cotton applicator tips; (**L**) bead sterilizer; (**M**) fiber optic light source.

While our experimental design involves sterile surgical techniques and basic suturing skills, this methodology does not require a microsurgical suite and can be adapted within many rodent facilities (Figure 1).

### 2.4. Experimental Timeline 

**Figure 2:**
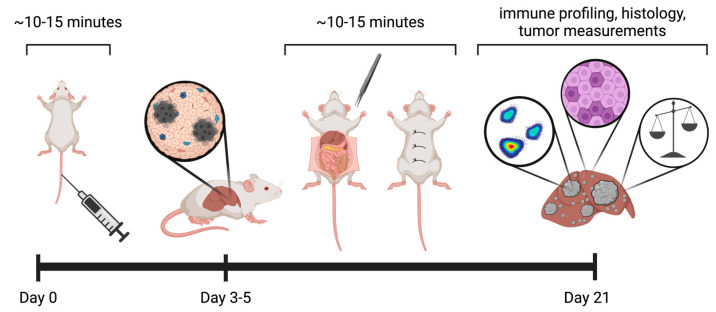
Timeline for vagotomy in mice with liver tumors. Procedure time per mouse is provided for tail vein and vagotomy procedures. Adult BALB/c mice receive a tail vein injection of A20 cells (see Section 3.1). Mice undergo a hepatic or sham vagotomy 3–5 days following tumor initiation; non-macroscopic tumors present (see Section 3.2). Experimental termination at 21 days following tumor initiation. Various endpoints, including tumorous liver measurements, histology, immune/metabolic profiling, and/or RNA-sequencing analyses, may be performed. In addition, this protocol can be adapted to include treatment strategies (e.g., immune checkpoint blockade) or systemic assessments (e.g., microbiota profiling via 16S rRNA-sequencing) (see [18]). Graphics, created in Biorender.com, provide representative models; precise surgical incision length is reported here in Figure 4.

After familiarization with the technique, hepatic and sham vagotomies can be performed within 15 min. Here, we provide a timeline for vagotomy in A20 tumor-bearing mice. All procedures are performed in adult mice, recommended 8–12 weeks of age. Tumor presence can be confirmed via standard hematoxylin and eosin (H&E) staining in a subset of mice (Figure 2).

## 3. Procedure

### 3.1. A20 Cell Preparation and Tail Vein Injection

Here, we provide an optional adapted methodology by which to perform hepatic vagotomy in a liver tumor model. Hepatic vagotomy can be performed in the researcher’s choice of tumor model, non-cancerous liver malignancy, and/or in healthy mice.

1. Grow A20 cells in RPMI + GlutaMAX cell media supplemented with 10% FBS, 1% penicillin/streptomycin, 14.3 M beta-mercaptoethanol, and 1 mM each of HEPES, NEAA, and sodium pyruvate. Cells should be utilized within 10 passages after thawing. A20 cells are a non-adherent, fast-growing cell line. Cell confluency should be assessed frequently via light microscope. We recommend passaging at a 1:5–1:10 ratio.
2. Prepare cells for tail vein injection. Count cells and suspend 1 × 10^6^ cells per 100 μL sterile PBS. Place cells in an Eppendorf tube and store on ice until injection.
3. Place mice into a procedural cage with heat lamp for 10–15 min to dilate veins. Restrain mice and sterilize tails with alcohol wipes. Don sterile gloves prior to the procedure. Inject tumor cells with a sterile 29 G syringe into the mouse lateral tail vein. Detailed tail vein injection procedures have been previously reported by our group [18,42] and others [see online standard operating procedures from University of British Columbia and University of California San Francisco, Intravenous Tail Vein Injections in the Adult Mouse and Lateral Tail Vein Injection in Mice and Rats, respectively].

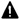
Additional notes: Cell culture kinetics will influence subsequent tumor burden. Cells collected in the growth phase will ensure rapid tumor growth. Prepare cells immediately prior to usage. As some samples may be lost during syringe preparation, we recommend preparing enough cells for two additional mice. To prevent cell clumping, vortex the Eppendorf tube with A20 cells prior to tail vein injection. If injecting for a large experiment, split samples into two vials and maintain one in ice during injections of the first set of mice. To avoid potential variability due to injection and/or time-to-inject, randomize mice following tail vein injection. Cells purchased from ATCC are authenticated and tested for mycoplasma and murine pathogens; the authors recommend regular molecular and biological testing via PCR to ensure purity and non-contamination of laboratory cell stocks.

### 3.2. Sham and Hepatic Vagotomy

**Figure 3:**
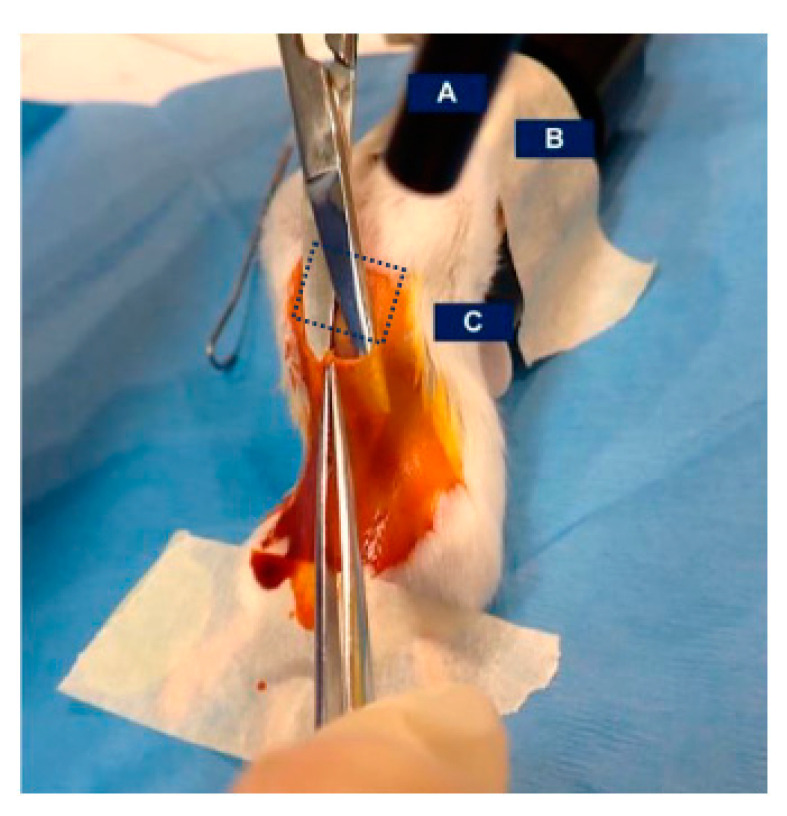
Mouse set-up during sham or hepatic vagotomy procedure: (**A**) fiberoptic light (off); (**B**) mouse arms taped to nose cone; and (**C**) separating the skin from the underlying peritoneal/muscular layer. Dotted square showing location of the underlying xiphoid process.

1. Perform perioperative analgesic per institutional requirements. Set-up surgical arena (see Figure 1), including staples and sufficient 5-0 vicryl sutures.
2. Anesthetize animals in 2.5% isoflurane chamber. If mice have not been shaved, remove fur from the surgical field (shave from pelvis to xiphoid process). As anesthetized mice will not blink, generously swab eyes with lubricating gel.
3. To maintain body heat, place mouse on a heated surgical table or place a heating pad under sterile draping. Use a nose cone system to maintain anesthesia.
4. Utilize surgical paper tape to prevent jostling the mouse. Attach the arms overhead to the isoflurane nosecone keeping the abdomen taut. Sharply pinch the hind paws to ensure an appropriate anesthetic plane prior to surgery.
5. Don loupes, sterile gloves, and gowning prior to surgical procedures.
6. Apply a generous swabbing of with 10% povidone–iodine solution followed by 70% ethanol and repeat twice more for a total of three administrations before surgery. Utilize autoclaved instruments. Maintain sterility via bead sterilization throughout procedure. Note: use a fresh set of sterilized tools per every five animals or for animals from a different cage. Place sterile draping over the surgical field. Use forceps to lift the skin and make a small incision at the base of the xiphoid process. Separate the skin from the underlying peritoneal layer and make a vertical incision from the xiphoid base to the lower abdomen (Figure 3).
7. Cut the underlying peritoneal/muscular layer along the linea alba, following a vertical incision.

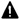
Additional notes: If utilizing scissors, keep the scissor tips pointed away from internal organs. Minor bleeding from the skin/peritoneal incision is expected and typically coagulates rapidly. Dabbing with a silver nitrate stick may provide rapid cauterization.

**Figure 4:**
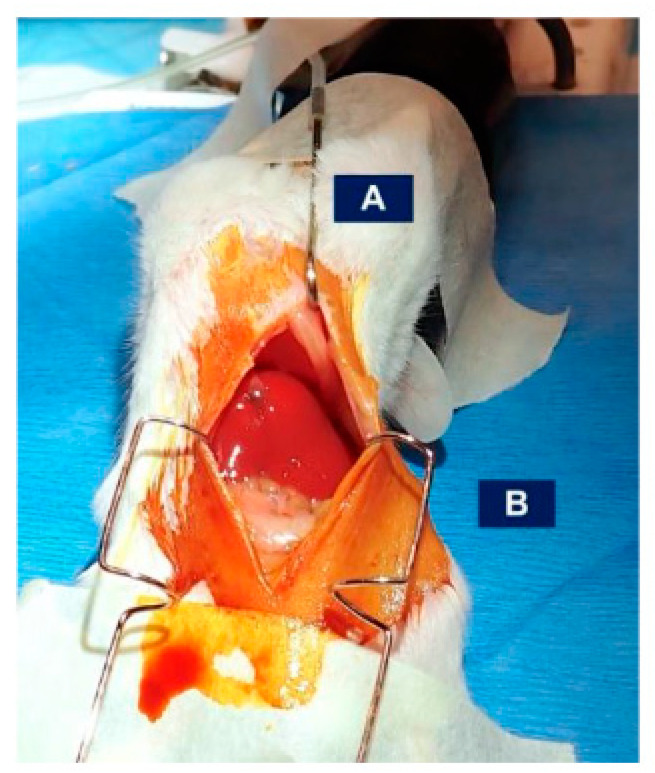
Retractor set-up for hepatic and sham vagotomy procedure using (**A**) a blunt hook retraction adjacent to the xiphoid process and (**B**) Kratz-Barraquer ocular-style retractor in the abdominal cavity with only tips inserted. Retractors are secured with paper tape. Prior to retraction, a vertical incision was performed along the linea alba.

**Figure 5:**
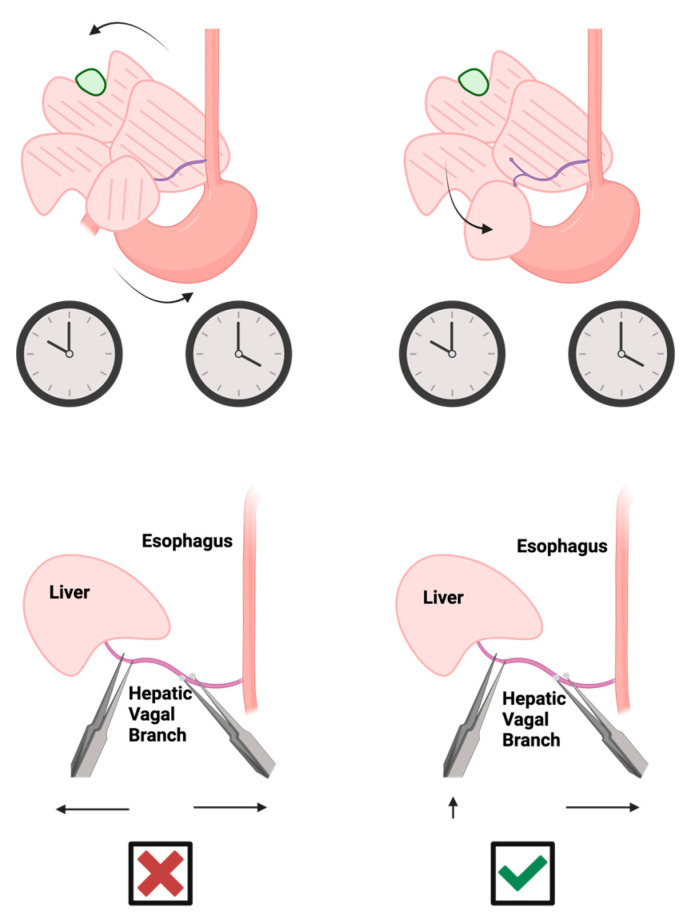
Hepatic vagotomy procedure: (**Top**) flip liver lobes up and towards 10 o’clock against the diaphragm with saline-soaked cotton applicators while gently pulling downwards and towards 4 o’clock on the stomach and esophagus; (**Bottom**) upon confirmation of the common hepatic vagal branch, pull the caudate lobe downwards over the stomach/intestines and isolate the branch with blunted micro-forceps. Use forceps to shear the hepatic vagal branch, pulling it apart with blunted micro-forceps towards the esophagus to protect the liver. Graphical images were created with BioRender.com.

**Figure 6:**
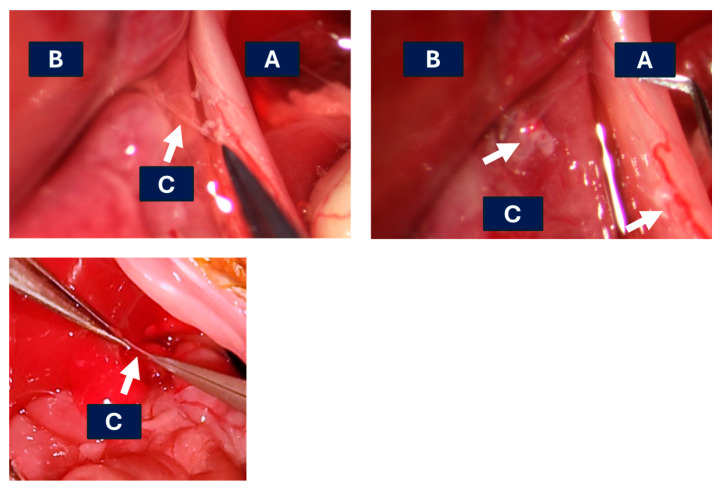
Representative image of the hepatic branch shear; (**A**) esophagus; (**B**) liver; (**C**) hepatic vagal branch. Top left: hepatic vagal branch identified with a white arrow. For ease of visualization, the hepatic nerve has been stripped of fat surrounding the common hepatic branch. Top right: vagotomized liver with white arrows indicating torn ends. Bottom: representative image of vagal branch shearing with micro-forceps (**C**) above the caudate lobe. Upper photos kindly provided by Dr. Lee Chedester.

8. Place retractors to maintain an open surgical field. Here, we utilize a combination of a Kratz-Barraquer retractor within the abdomen and a blunt retractor placed near the xiphoid process, forming a diamond-shaped surgical field. Use surgical tape to keep retractors in place (Figure 4). Note: to reduce incision size and stabilize the incision more easily, the Kratz-Barraquer retractors are inverted and only the tip is utilized during insertion.
9. Using saline-soaked cotton applicators, gently lift/flip the liver lobes in a counter-clockwise manner while gently pulling the esophagus and stomach in a clockwise manner (Figure 5).
10. Locate the common hepatic branch where it forks off the esophagus (under 5 cm) above the stomach. The common hepatic vagus enters the liver above the caudate lobe. To improve access to the branch, separate the caudate lobe, flipping it downward over the intestines.
11. Utilizing blunt micro-forceps, tease the vagus nerve away from the esophagus and liver. Note: this may cause minor bleeding from the liver capsule. Once the common hepatic branch is isolated, use a super fine tip micro-forcep and clamp down on the branch. Shear the nerve by pulling it apart. Maintain grip with the super-fine micro-forcep and pull apart with the blunt micro-forcep towards the esophagus to prevent laceration of the liver capsule or hepatic blood vessels (Figure 6). *For sham surgical controls, repeat steps 1–11, isolating, but not shearing, the hepatic branch.*
12. After the hepatic branch tears, maintain a tight grip of both forceps, allowing for coagulation, as the nerve runs alongside minor blood vessels.

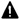
Additional notes: Only use saline-soaked cotton applicators when manipulating the organs (Step 9); use of dry cotton buds will produce adhesions on the liver surface and potentially break the fragile liver capsule. After flipping the liver upwards, it will lightly “stick” to the diaphragm/peritoneal wall; avoid excess pressure on the liver. If minor bleeding occurs after the vagotomy, a dry cotton applicator can be utilized to staunch bleeding until coagulation (Steps 10–12). Only use a dry cotton applicator at the site of a bleed. Generously fill the abdominal cavity with sterile saline to further protect against the formation of internal adhesions.

**Figure 7:**
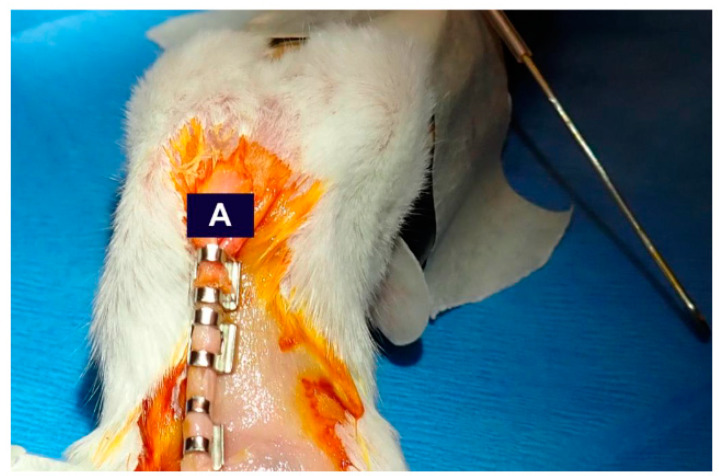
Mouse staples after surgery. After retractor removal (blunt retractor visible) and suturing (Step 14), (**A**) place 3–4 staple sutures to close the vertical incision.

13. After ensuring no active bleeds within the abdomen, use a sterile syringe to add ~0.5–1 mL of sterile saline, “packing” the abdomen and liver lobes with saline to prevent adhesion formation within internal organs.
14. Close the peritoneum with continuous or interrupted sutures with 5-0 VICRYL™.
15. Using forceps, pull the skin together and close with staples or clips (Figure 7).

16. Provide additional perioperative analgesic per institutional requirements.
17. Place the animal in a postoperative cage with a heat lamp for monitoring. Animals should begin to move and climb around the cage within 3–10 min of the procedure.

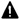
Additional notes: Do not use cold saline to pack the intestines. If stored at 4 °C, warm saline to room temperature or place in a heat bath. If the procedure room has multiple nose cones available, one surgeon can perform the vagotomy (Steps 1–12) and another can close the abdomen (Steps 13–15). Ideally, surgical procedures will be performed in a two-member team. This will shorten procedure time across large experiments. Use multiple clips and ensure no loose clips are placed on the skin. Mice will bite loose clips and gnaw at accessible surgical sutures, potentially leading to death. Animal death from improper vagotomy occurs rapidly and is likely the result of an internal bleed from the liver. We urge researchers to monitor animals for 30 min following surgery and daily throughout the first 72 h. Use of soft rodent chow during this timeframe is recommended. Skin staples/clips should be removed 10–14 days following surgery.

## 4. Expected Results and Conclusions

We have not observed changes in mouse body weight, liver weight, or histological features (H&E staining) following hepatic vagotomy, utilizing mice that underwent hepatic vagotomy by Charles River Laboratories surgical services as reported [18]. Moreover, we previously showed that hepatic vagotomy prior to cancer initiation reduces liver tumors burden in primary liver cancer, including an intrahepatic injection model described in Brown et al., 2018 [18,45].

**Figure 8:**
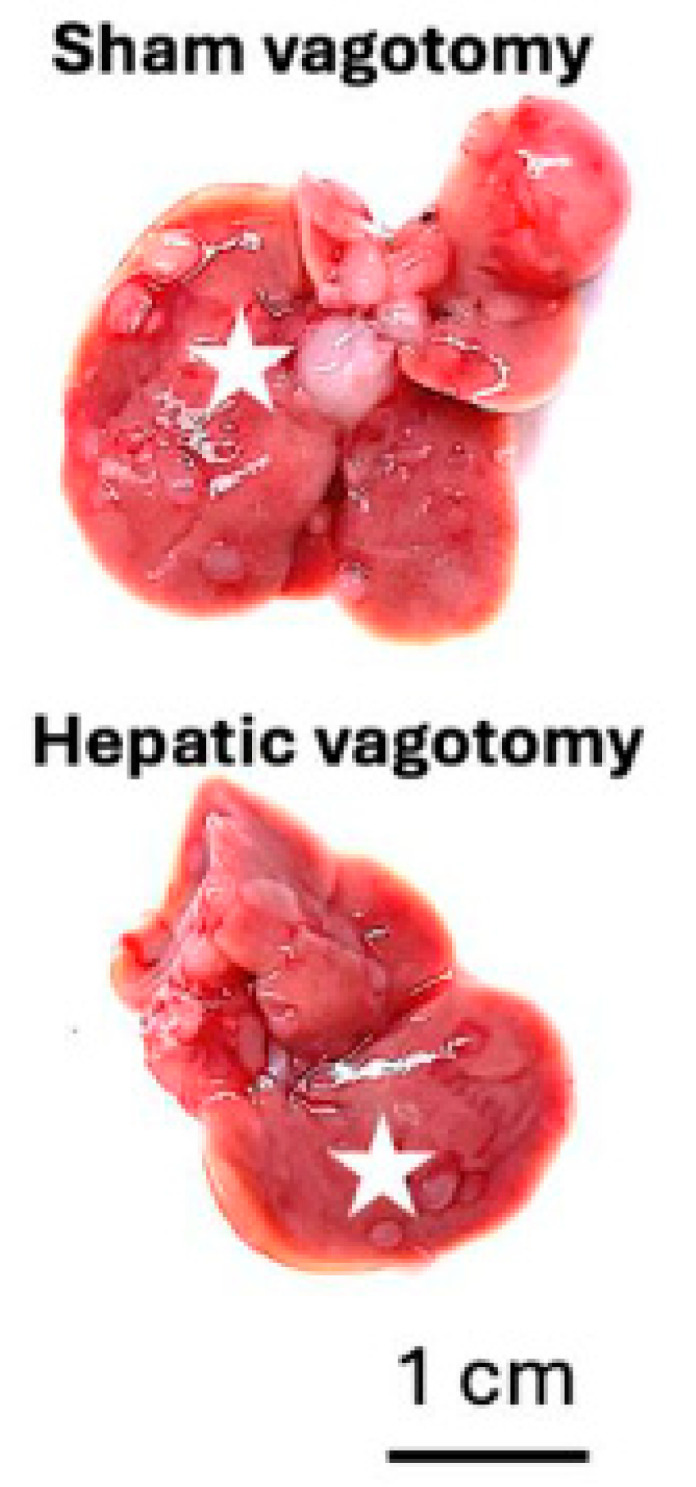
Numerous A20 metastatic tumors are present 21 days following tail vein injection. Representative livers from sham (**top**) and hepatic vagotomized (**bottom**) mice. White stars highlight metastatic tumor-abundant regions.

To our knowledge, hepatic vagotomy procedures are not commercially available within North America by established mouse vendors (e.g., Charles River Laboratories, Jackson Laboratories). Here, we provide a detailed methodology of hepatic branch vagotomy and a sham surgical procedure. In contrast to earlier reports, we show that vagotomy can be performed following tumor initiation. This amended protocol utilizes a model of liver metastasis with a lymphoma A20 cell line (Figure 8). We selected this model as tumor initiation occurs via tail vein injection, rather than via surgical intrahepatic injection, thereby avoiding two major abdominal surgeries. The A20 model results in diffuse, punctate tumors. We observed fewer metastatic tumors in the hepatic model.

While our adapted protocol does not include a primary liver cancer model, we suggest that this model could be adapted for various genetic or inducible cancer models, as described by our colleagues [46], additional metastatic models beyond A20 cells, and non-cancer hepatic malignancies. Tumorous liver weight and histology can provide relatively rapid assessment of tumor burden at the experimental endpoint. Liver tissues can be further assessed via metabolic and immune profiling techniques.

The liver is the primary site of cancer metastasis, with over one quarter of all metastatic cancer developing within the liver. Both metabolic disorders and a highly tolerogenic immune landscape contribute to poor treatment outcomes in liver tumors [19,47,48]. Ongoing systems biology studies have revealed the dynamic, bidirectional interaction of vagal nerves with the immune system, metabolic processes, and tumor development [14,26,27,49]. How vagal nerves shape liver cancer metastasis and influence treatment efficacy, including emerging immunotherapies, remains relatively unknown. This technique provides a valuable tool for incorporation into systemic nerve–liver mouse studies and furthers the emerging cancer neuroscience field.

## Data Availability

No new data were created. Any questions should be addressed to Tim F. Greten.
